# Finite-Time Thermodynamic Modeling and a Comparative Performance Analysis for Irreversible Otto, Miller and Atkinson Cycles

**DOI:** 10.3390/e20010075

**Published:** 2018-01-19

**Authors:** Jinxing Zhao, Fangchang Xu

**Affiliations:** School of Mechanical Engineering, University of Shanghai for Science and Technology, 516 Jungong Rd., Shanghai 200093, China

**Keywords:** Atkinson and Miller cycles, over-expansion cycle, LIVC, energy losses, cycle performances, finite-time thermodynamics

## Abstract

Finite-time thermodynamic models for an Otto cycle, an Atkinson cycle, an over-expansion Miller cycle (M1), an LIVC Miller cycle through late intake valve closure (M2) and an LIVC Miller cycle with constant compression ratio (M3) have been established. The models for the two LIVC Miller cycles are first developed; and the heat-transfer and friction losses are considered with the effects of real engine parameters. A comparative analysis for the energy losses and performances has been conducted. The optimum compression-ratio ranges for the efficiency and effective power are different. The comparative results of cycle performances are influenced together by the ratios of the energy losses and the cycle types. The Atkinson cycle has the maximum peak power and efficiency, but the minimum power density; and the M1 cycle can achieve the optimum comprehensive performances. The less net fuel amount and the high peak cylinder pressure (M3 cycle) have a significantly adverse effect on the loss ratios of the heat-transfer and friction of the M2 and M3 cycles; and the effective power and energy efficiency are always lower than the M1 and Atkinson cycles. When greatly reducing the weights of the heat-transfer and friction, the M3 cycle has significant advantage in the energy efficiency. The results obtained can provide guidance for selecting the cycle type and optimizing the performances of a real engine.

## 1. Introduction

Internal combustion engines have achieved great success especially in the automotive industry. A diesel engine can achieve a higher thermal efficiency, but with more NOx and soot emissions and higher cost and NVH (Noise, Vibration and Harshness (NVH)) level compared with an Otto cycle engine. The cost of Otto cycle engines is less, but the thermal efficiency is limited because of low CR (compression ratio (CR)). The compression and expansion strokes are the same for an Otto cycle engine; and the increment of the CR of an Otto cycle engine is restricted by the knock [1]. Therefore, a great improvement of the thermal efficiency for Otto cycle engines is difficult. The attention on Atkinson and Miller cycle engines is greater and greater in recent years because of the high level of thermal efficiency and special applications in hybrid vehicles [2,3,4].

The ACE (Atkinson cycle engine (ACE)) is a type of full expansion internal combustion engine invented by James Atkinson in 1882 [5]. The original ACE used a complex linkage mechanism to make the expansion stroke greatly longer than the compression stroke aimed at achieving a high thermal efficiency. In addition to the ACE, an MCE (Miller cycle engine (MCE)) is another type of over-expansion cycle engine. In 1947, an American engineer named Ralph Miller patented an MCE [6,7]. In the original MCE, a compression control valve was placed in the cylinder head. A servo mechanism operated by the inlet manifold pressure controls the lift of the compression control valve during part of the compression stroke to release partial air from the cylinder to the exhaust manifold. In this way, the effective CR decreases and is less than the expansion ratio. The original MCEs were mainly used in ships or stationary power-generating plants aimed at reducing the NOx emissions. In recent years, the ACEs realized through variable valve timing technology have been widely employed in hybrid cars. With the variable valve timing mechanism, an LIVC (late intake valve closure (LIVC)) operation can be carried out to make the expansion stroke longer than the compression stroke. The MCEs can be realized with the same method. However, the ACEs are naturally aspirated, while the MCEs are generally equipped with a super-/turbocharger [6,7]. Thus, a modern MCE is essentially the same as a modern ACE [8,9].

There are three types of over-expansion cycles, which are the full expansion Atkinson cycle, the over-expansion Miller cycle and the LIVC Miller cycle [4]. The full expansion Atkinson cycle is realized by fully expanding to the barometric pressure to draw the full working potential of in-cylinder working fluids. The over-expansion Miller cycle is realized by making the expansion stroke longer than the compression stroke. The LIVC Miller cycle is realized by an LIVC (or EIVC) operation based on a normal Otto cycle. The expansion stroke is the same as the Otto cycle, while the effective compression stroke shortens. In this simple way, an over-expansion cycle is realized. In those previous publications, many investigations have been conducted for over-expansion Miller cycles. In [10], Gonca conducted a performance analysis for an air-standard irreversible dual-Miller cycle with an LIVC version. However, the Miller cycle is still essentially an over-expansion type; and the modeling process is basically the same as in the other investigations. The over-expansion cycle was realized by way of extending the expansion stroke; and the mixture backflow process because of the LIVC operation was not considered.

Many investigations on the thermodynamic analyses, comparisons and optimizations for the ACEs and MCEs have been conducted based on the FTT (finite-time thermodynamics (FTT)) [11,12,13,14,15,16,17,18,19,20,21,22,23,24,25] or the classical thermodynamics [26,27]. Ge and Chen et al. investigated the performances of the Atkinson cycles [12,13] and an irreversible Miller cycle [14] by using the FTT method, respectively. In [12], the efficiency of an Atkinson cycle engine at the maximum power density was studied. The results showed that the efficiency at the maximum power density was greater than that at the maximum power. In [13], an Atkinson cycle was analyzed with the consideration of the heat-transfer and friction-like losses; and some relations such as the power output and the CR were derived. The results showed that the heat-transfer and friction-like losses had significant negative influences in the cycle performances and should be taken into account in building the FTT models. Al-Sarkhi et al. evaluated the performances of the Miller cycles under various specific-heat models such as constant, linear and forth-order polynomial [15,16]. Lin and Hou et al. performed some studies on the performances and influences of the heat loss, friction and variable specific-heats for Atkinson and Miller cycles [17,18,26]. In [26], Wang and Hou conducted a performance analysis and comparison based on MP (maximum power (MP)) and MPD (maximum power density (MPD)) conditions for an Atkinson cycle by using the classical thermodynamic method. In [17,18], the effects of the heat loss, derived as a percentage of the fuel’s energy, on the performances of an Atkinson cycle and a Miller cycle were investigated, respectively. The influences of some other engine parameters such as the friction and intake temperature on the performances were also studied. Gonca et al. carried out a thermodynamic analysis on the performances of a dual-Atkinson cycle considering the heat-transfer with the influences of the design and operation parameters, friction, temperature-dependent specific-heat and internal irreversibilities based on the classical thermodynamics and the FTT methods, respectively [27]. The influences of important engine design and operation parameters such as the engine speed, stroke length and CR on the performances were deeply analyzed. Lin et al. [19] applied the FTT method to analyze the performances of a free piston MCE considering heat-transfer, friction and internal irreversibilities. They stated that the heat-transfer and friction losses had negative effects on the performances. Ebrahimi R investigated the effects of mean piston speed, equivalence ratio and cylinder wall temperature on the performances of an ACE considering the heat-transfer, friction and internal irreversibilities [20]. Dobrucali E showed that the engine design and running parameters had significant effects on the performances of an Otto–Miller cycle engine; and the friction and inlet temperature had a negative effect on the performances [21]. Mousapour A et al. analyzed the effects of key engine design parameters such as the minimum and maximum cycle temperatures and CR on the power output and the first and second law efficiencies of an irreversible Miller cycle considering heat-transfer, friction and internal irreversibilities [22]. Ust Y et al. conducted a performance analysis and optimization based on exergetic performance criterion, total exergy output and exergy efficiency for an irreversible dual-Miller cycle cogeneration system with finite-rate of heat-transfer, heat-leak and internal irreversibilities; and the optimum design parameters such as the cut-off ratio and Miller cycle ratio that maximize the exergy output and exergetic performance were investigated [23]. Curto-Risso PL et al. [24] justified that an FTT model of an Otto cycle with irreversibilities from friction, heat-transfer through the cylinder walls and internal irreversible losses was capable of qualitatively reproducing the performance results of a real spark ignition heat engine by comparing to a quasi-dimensional simulation model. Moreover, they also showed that the inclusion of the corresponding polynomial fit in the FTT model for some key parameters such as the internal irreversibility was necessary to precisely reproduce the results of the quasi-dimensional simulation. In [25], Curto-Risso PL et al. further studied the effect of spark advance, fuel ratio and cylinder internal wall temperature on the power output and efficiency of spark ignition engines by combining quasi-dimensional simulations and FTT analysis. The optimal dependence on rotational speed of these operation parameters was achieved in order to maximize efficiency under any power output. Some studies specially conducted the comparative analyses and performance optimizations among different thermodynamic cycles such as Otto, Miller and Atkinson [14,28,29,30]. Hou [30] compared the performances of Atkinson and Otto cycles. The results illustrate that the Atkinson cycle has a greater work output and a higher thermal efficiency than the Otto cycle at the same operation condition. The compression ratios maximizing the work of the Otto cycle are always higher than the Atkinson cycle at the same operation condition. Ge et al. [14] analyzed the performances of an air-standard Miller cycle with the considerations of heat-transfer and friction loss. Furthermore, they compared the performances of an Otto cycle, a Miller cycle and an Atkinson cycle. It is found that the Atkinson cycle can achieve the highest output work and cycle efficiency while those for the Otto cycle are the lowest at the same running condition. Gonca et al. [29] conducted comprehensive performance analyses and comparisons for air-standard thermodynamic cycle engines considering internal irreversibility based on some criteria such as MP. The thermodynamic cycles include the Otto cycle, dual-Atkinson, Otto–Atkinson, dual-Miller, etc. The results show that the diesel cycle has the greatest MPD and the Atkinson cycle has the least MPD; the MP of the diesel cycle is the minimum, and the MP of the Atkinson cycle is the maximum. However, the Miller cycle has the best values in terms of thermal efficiency, as the diesel cycle has the lowest values. In [28], Gonca performed comparative performance analyses of the irreversible OMC engine, diesel Miller cycle engine and dual Miller cycle engine based on the MP, MPD and MEF criteria.

In most of the previous publications related to the FTT methods, the losses for the heat-transfer and friction were not considered or treated as particularly simple forms. The heat loss through the cylinder wall was assumed to be proportional to the difference of the average temperature between the working fluids and cylinder wall, or as a percentage of the energy of the fuel in the cylinder. The heat-transfer coefficients did not change with the changes of the engine design parameters and cycle conditions. Furthermore, for the friction lost power, all only considered the viscous friction with respect to the mean piston speed; and did not consider the effects of important engine parameters such as the cylinder pressure and the stroke length. In [31], Zhao et al. demonstrated that the engine design parameters and cycle conditions had significant effects on the heat-transfer coefficient, thus the heat losses through the cylinder wall; and the boundary friction primarily related to the cylinder pressure also had non-negligible influences on the engine cycle performances. In comparative analyses of different thermodynamic cycles as in [14,29], the comparative results and change characteristics for the energy losses and performances may have significant differences from real engine processes if the heat-transfer and friction are not considered or only handled with simple relations. As a result, it is not capable of precisely clarifying the respective advantages and disadvantages of each thermodynamic cycle studied; and the comparative analysis studies may lose real meaning and cannot give correct guidance for the cycle selection and performance optimization of real engines.

The objective of this paper is to carry out a comparative analysis on the energy losses and performances for an Atkinson cycle, an Otto cycle, an over-expansion Miller cycle, an LIVC Miller cycle realized through late intake valve closure and an LIVC Miller cycle with CCR (constant compression ratio (CCR)) in order to clarify their respective advantages and disadvantages; and expected to provide more correct guidance for the cycle selection and performance optimization of practical engines. For this purpose, the FTT models for these cycles are first developed with the irreversible losses from the heat-transfer to the cylinder wall and the friction. The influences of the cycle conditions, the geometrical parameters and running variables on the heat-transfer and friction are all considered. Especially, thermodynamic models of the two LIVC Miller cycles different from the previous over-expansion versions are originally developed considering the process of the mixture backflow. Then, a deep analysis and a comparative study for the energy losses and performances of these cycles are conducted; and some important findings have been achieved.

## 2. Cycle Models

**Figure 1:**
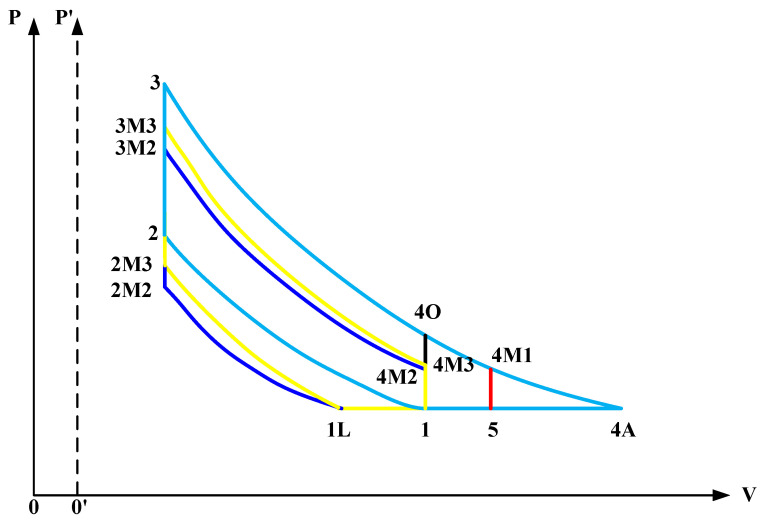
P-V diagrams for the Otto, Atkinson and Miller cycles.

Figure 1 shows the P-V diagrams for an Otto cycle, an over-expansion Miller cycle, an Atkinson cycle and two LIVC Miller cycles. All the cycles and the respective abbreviations have been listed in Table 1.

In the figure, 1-2-3-4O-1 is an Otto cycle, and 1-2-3-4A-1 is an Atkinson cycle process. The other three processes that are 1-2-3-4M1-5-1, 1-1L-2M2-3M2-4M2-1 and 1-1L-2M3-3M3-4M3-1 are an over-expansion Miller cycle (M1), an LIVC Miller cycle (M2) and an LIVC Miller cycle with CCR (M3), respectively.

1-1L is an LIVC process with a charge backflow. 1-2, 1L-2M2 and 1L-2M3 are reversible adiabatic compression processes. 2-3, 2M2-3M2 and 2M3-3M3 are isochoric heat-addition processes. 3-4A, 3-4O, 3-4M1, 3M2-4M2 and 3M3-4M3 are reversible adiabatic expansion processes. 4O-1, 4M3-1, 4M2-1 and 4M1-1 are isochoric heat rejection processes. 4A-1 and 5-1 are isobaric heat rejection processes. 

For the process 1-1L, intake charges are not compressed because of the LIVC and mixture backflow; and pressure and temperature of the intake charges remain nearly constant. In this process, the net mass of intake charges and real compression ratio decreases. By defining:

(1) $$r_{m2}=r_{c\_M2}/r_{c},$$

maintaining constant equivalence ratio and applying the ideal gas equation:

(2) $$pV=mR_{g}T,$$

the net mass flow rate of the intake charges for the M2 cycle can be obtained:

(3) $${\dot{m}}_{c\_M2}=r_{m2}{\dot{m}}_{c}$$

where $r_{c}=V_{1}/V_{2}$ is the compression ratios of the cycles shown in Figure 1 except the M2 cycle; ${\dot{m}}_{c}$ is the intake mass flow rate for the Otto, M1 Miller and Atkinson cycles; $r_{c\_M2}=V_{1L}/V_{2M2}$ is the compression ratio of the M2 cycle; and $r_{m2}$ is the ratio of $r_{c\_M2}$ to $r_{c}$. 

As for the M3 cycle, the net mass flow rate of the intake charges is the same as that of the M2 cycle since their start points of compression processes are the same ($r_{m3}=r_{m2}$). The compression ratio is maintained constant by way of adjusting combustion chamber volume according to the LIVC value. As shown in Figure 1, the $P^{\prime }-0^{\prime }$ axis in the form of a dashed line is specially set for the M3 cycle and indicates the decrement of the combustion chamber volume in order to maintain a constant CR. In this situation, the relevant parameters for the M3 cycle are written as:

(4) $$r_{c\_M3}=r_{c}$$

(5) $$r_{e\_M3}=\frac{r_{c}}{r_{m3}}$$

where $r_{c\_M3}$ and $r_{e\_M3}$ are the compression ratio and the expansion ratio of the M3 cycle, respectively.

Displacement volumes are all the same for the Otto, M2 and M3 cycles; and can be computed as:

(6) $$V_{d}=\frac{1}{4}\pi B^{2}L$$

where *B* is the cylinder bore and *L* is the stroke length for the Otto, M2 and M3 cycles. 

Piston strokes and displacement volumes for the other cycles shown in Figure 1 can be computed as:

(7) $$L_{M1}=\frac{r_{m1}r_{c}-1}{r_{c}-1}L$$

(8) $$V_{d\_M1}=\frac{1}{4}\pi B^{2}\frac{(r_{e\_M1}-1)L}{r_{c}-1}$$

where:

(9) $$r_{e\_M1}=\;r_{m1}r_{c}$$

is the expansion ratio of the M1 cycle; and $r_{m1}$ is the ratio of $r_{e\_M1}$ to $r_{c}$. The Otto and Atkinson cycles are two special conditions of the M1 cycle; and the range for the $r_{e\_M1}$ is:

(10) $$r_{c}\leq r_{e\_M1}\leq \frac{T_{4A}}{T_{1}}r_{c}$$

The piston stroke length and displacement volume of the Atkinson cycle can be obtained by substituting the expansion ratio $r_{e\_At}$ ($=\frac{T_{4A}}{T_{1}}r_{c}$) into Equations (7) and (8).

In a real ICE, the cylinder gas temperature changes greatly in the cycle processes; and the specific-heat of working fluids also accordingly changes with the cylinder gas temperature [32,33]. It is assumed that the working fluids are the ideal gas; and a temperature-dependent specific-heat model with second order form for constant pressure processes can be written as [21]:

(11) $$C_{p}=2.506\times 10^{-11}T^{2}+1.454\times 10^{-7}T^{1.5}-4.246\times 10^{-7}T+3.162\times 10^{-5}T^{0.5}+1.3303\;-1.512\times 10^{4}T^{-1.5}+3.063\times 10^{5}T^{-2}-2.212\times 10^{7}T^{-3}$$

Accordingly, a variable specific-heat model for constant volume processes can be achieved by the following relation:

(12) $$C_{v}=C_{p}-R_{g}$$

where $R_{g}=0.287\;\mathrm{kJ}/\mathrm{kgK}$ is the state constant of the ideal gas. 

The input rate of total heat energy in the heat addition processes shown in Figure 1 can be computed as:

(13) $$Q_{T}={\dot{m}}_{f}Q_{LHV}$$

where ${\dot{m}}_{f}$ and $Q_{LHV}$ are the mass flow rate and the lower heat value of the fuel, respectively. The mass flow rate of the fuel injected into the cylinder can be computed as:

(14) m˙f=ϕm˙a(m˙am˙f)s−1

Then, the total working fluids flowing into the cylinder can be computed as:

(15) m˙c=m˙a+m˙f=m˙f(ϕ−1(m˙am˙f)s+1)

where ϕ and (m˙am˙f)s are the equivalence ratio and the stoichiometric air-fuel ratio, respectively. The rate of heat energy added to the working fluids by combustion during the process 2-3 can be written as:

(16) $${\dot{Q}}_{in}={\dot{m}}_{c}\int_{T_{2}}^{T_{3}}C_{v}dT$$

Similarly, the heat added to the working fluids per second for the process 2M2-3M2 and 2M3-3M3 can be computed by substituting $T_{2}$ and $T_{3}$ in Equation (16) with $T_{2M2}$, $T_{2M3}$ and $T_{3M2}$, $T_{3M3}$, respectively.

The heat rejection rate into the environment during the process 4M1-5 can be computed as:

(17) $${\dot{Q}}_{out\_4M1-5}={\dot{m}}_{c}\int_{T_{5}}^{T_{4M1}}C_{v}dT$$

The heat rejection rate into the environment during the process 5-1 can be computed as:

(18) $${\dot{Q}}_{out\_5-1}={\dot{m}}_{c}\int_{T_{1}}^{T_{5}}C_{p}dT$$

(19) $$T_{5}=r_{e\_M1}T_{1}$$

Similarly, the heat rejection rate into the environment during the process 4M2-1 and 4M3-1 can be respectively computed as:

(20) $${\dot{Q}}_{out\_4M2-1}={\dot{m}}_{c}\int_{T_{1}}^{T_{4M2}}C_{v}dT$$

(21) $${\dot{Q}}_{out\_4M3-1}={\dot{m}}_{c}\int_{T_{1}}^{T_{4M3}}C_{v}dT$$

According to [34], the heat-transfer rate through cylinder walls to coolant can be computed as:

(22) $${\dot{Q}}_{tr}=h_{c}A_{cc}\left(T_{m}-T_{w}\right)$$

where the $A_{cc}$, $T_{m}$ and $T_{w}$ are the approximate surface area of the combustion chamber, mean in-cylinder gas temperature and cylinder wall temperature, respectively; and the $h_{c}$ is the heat-transfer coefficient, which can be computed as:

(23) $$h_{c}=CB^{-0.2}p^{0.8}w^{0.8}T_{m}{}^{-0.55}$$

where *C* is a constant, p is instantaneous cylinder pressure and *w* is average in-cylinder charge velocity, which can be written as [34]:

(24) $$w=\left[C_{1}{\bar{S}}_{p}+C_{2}\frac{V_{d}T_{r}}{P_{r}V_{r}}(p-P_{m})\right]$$

where $C_{1}$ and $C_{2}$ are constants; ${\bar{S}}_{p}$ is average piston speed; $P_{r}$, $V_{r}$, $T_{r}$ are the pressure, volume and temperature at the reference state; and $P_{m}$ is the cylinder pressure at the same crank angle as p as the engine works in motored conditions. It is shown in Figure 1 that $P_{m}$ is equal to the cylinder pressure at the end of compression stroke under ideal conditions. As shown in Figure 1, select the points 2, M2 and M3 as reference states for the Otto (M1 and Atkinson), M2 and M3 cycles, respectively. The instantaneous cylinder pressure in Equations (23) and (24) is substituted by the respective mean value. Then, the corresponding values in Equations (23) and (24) for the Otto, M1 and Atkinson cycles can be changed as:

(25) $$P_{m}=P_{2}$$

(26) p=(P3+P2)2

(27) $$P_{r}=P_{2}$$

(28) $$V_{r}=V_{2}=\frac{1}{4}\pi B^{2}\frac{L}{r_{c}-1}$$

(29) $$T_{r}=T_{2}$$

The corresponding values for the M2 and M3 cycles are similar as that for the Otto cycle (or M1 and Atkinson cycles), which can be achieved by replacing $P_{2}$, $P_{3}$ and $T_{2}$ with ($P_{2M2}$, $P_{2M3}$), ($P_{3M2}$, $P_{3M3}$) and ($T_{2M2}$, $T_{2M3}$), respectively. The $V_{r}$ of the M2 cycle is the same as that in the Equation (28); the $V_{r}$ of the M3 cycle is less than the other cycles and can be expressed as:

(30) $$V_{r\_M3}=V_{2\_M3}=\frac{1}{4}\pi B^{2}\frac{r_{m3}L}{r_{c}-r_{m3}}$$

The combustion chamber surface area for the Otto, M1, Atkinson and M2 cycles can be approximately estimated as:

(31) $$A_{cc}=\frac{1}{2}\pi B^{2}+\pi BL\frac{1}{r-1}$$

Additionally, the combustion chamber surface area for the M3 cycle can be expressed as:

(32) $$A_{cc\_M3}=\frac{1}{2}\pi B^{2}+\pi BLr_{m3}\frac{1}{r_{c}-r_{m3}}$$

The rate of heat energy added into working fluids can also be computed as:

(33) $${\dot{Q}}_{in}=\eta_{com}{\dot{m}}_{f}Q_{LHV}-h_{c}A_{cc}\left(T_{m}-T_{w}\right)$$

in which the combustion efficiency $\eta_{com}$ and mean gas temperature $T_{m}$ can be written as [20]:

(34) $$\eta_{com}=-1.44738+4.18581\phi^{-1}-1.86876\phi^{-2}$$

(35) $$T_{m}=\frac{T_{2}+T_{3}}{2}$$

The energy conservation equation for a close system can be written as:

(36) $$TdS=\delta Q_{w}+pdV=C_{v}dT+pdV=C_{p}dT-Vdp$$

where the $\delta Q_{w}$ represents the increased amount of heat energy as the temperature changes by dT. Apply the ideal gas state equation to the Equation (36), and divide *T* for both sides; then, the following equation can be achieved by integrating the Equation (36):

(37) $$S_{j}-S_{i}=\int_{T_{i}}^{T_{j}}\frac{C_{v}}{T}dT+R_{g}\ln\frac{V_{j}}{V_{i}}=\int_{T_{i}}^{T_{j}}\frac{C_{p}}{T}dT-R_{g}{\ln p|}_{p_{i}}^{p_{j}}=0$$

The following two equations can be achieved for the processes 1-2 of the Otto, M1 and Atkinson cycles:

(38) $$\int_{T_{1}}^{T_{2}}\frac{C_{v}}{T}dT=R_{g}\ln r_{c}$$

(39) $$\int_{T_{1}}^{T_{2}}\frac{C_{p}}{T}dT=R_{g}\ln\frac{P_{2}}{P_{1}}$$

The corresponding equations for the M2 and M3 cycles can be obtained by way of replacing $T_{2}$, $P_{2}$, $r_{c}$ in Equations (38) and (39) with $T_{2M2}$, $P_{2M2}$, $r_{c\_M2}$ and $T_{2M3}$, $P_{2M3}$, $r_{c}$, respectively. 

The following two equations can be achieved for the process 3-4M1:

(40) $$\int_{T_{4M1}}^{T_{3}}\frac{C_{v}}{T}dT=R_{g}\ln r_{e\_M1}$$

(41) $$\int_{T_{4M1}}^{T_{3}}\frac{C_{p}}{T}dT=R_{g}\ln\frac{P_{3}r_{e\_M1}T_{1}}{T_{4\_M1}}$$

The corresponding equations for the Otto and Atkinson cycles can be obtained by replacing $T_{4M1}$, $r_{e\_M1}$ in Equations (40) and (41) with $T_{4O}$, $r_{c}$ and $T_{4A}$, $r_{e\_AT}$, respectively. The corresponding equations for the M2 and M3 cycles can be obtained by replacing $T_{3}$, $T_{4M1}$, $r_{e\_M1}$ in Equation (40) with $T_{3M2}$, $T_{4M2}$, $r_{c}$ and $T_{3M3}$, $T_{4M3}$, $r_{e\_M3}$, respectively. The corresponding equations for the M2 and M3 cycles can be obtained by setting $r_{e\_M1}=1$ and replacing $T_{3}$, $T_{4M1}$, $P_{3}$ in Equation (41) with $T_{3M2}$, $T_{4M2}$, $P_{3M2}$ and $T_{3M3}$, $T_{4M3}$, $P_{3M3}$, respectively.

The studies in [31] show that the total friction losses include the losses from the boundary friction and the viscous friction. The boundary friction is mainly influenced by the engine design parameters and the peak cylinder pressure; and the viscous friction is mainly influenced by the engine design parameters and the average piston speed. Therefore, the *FMEP* (friction mean effective pressure (*FMEP*)) under boundary conditions can be written as:

(42) $${\left(FMEP\right)}_{boundary}=a\times \frac{L}{B^{2}}\times P_{\max}$$

where the coefficient *a* can be calibrated to consider the pressure-affected friction in the camshaft and crankshaft system of a real engine. 

Additionally, the viscous *FMEP* can be expressed by:

(43) $${\left(FMEP\right)}_{hydrodyn}=b\times \frac{A_{p,eff}}{LB^{2}}\times \bar{S_{p}}$$

where $A_{p,eff}$ represents the effective piston-skirt area in contact with cylinder liner. The coefficient *b* can be calibrated to consider speed-dependent friction in the camshaft and crankshaft system of a real engine. 

Then, the lost power from friction can be computed as [34]:

(44) $$P_{f}(kW)=\frac{FMEP(kpa)\times V_{d}(dm^{3})\times N(r/s)}{2\times 10^{3}}$$

whereFMEP is the sum of the ${\left(FMEP\right)}_{boundary}$ and ${\left(FMEP\right)}_{hydrodyn}$; and the engine rotational speed *N* can be written as:

(45) $$N=\bar{S_{p}}/2L$$

Therefore, the effective output power for any cycle in Figure 1 can be written as:

(46) $$P_{e}={\dot{Q}}_{in}-{\dot{Q}}_{out}-P_{f}$$

Accordingly, the energy efficiency and the power density can be computed as follows:

(47) $$\eta =\frac{{\dot{Q}}_{in}-{\dot{Q}}_{out}-P_{f}}{{\dot{Q}}_{T}}$$

(48) $$P_{d}=\frac{P_{e}}{V_{d}}$$

when $r_{c}$ and $T_{1}$ are known and $T_{2}$ and $P_{2}$ can be achieved by solving Equations (38) and (39) for the Otto, M1 and Atkinson cycles. Similarly, when $r_{c}$, $r_{m2}$ and $T_{1L}$ are known, $T_{2M2}$, $T_{2M3}$, $P_{2M2}$ and $P_{2M3}$ can be obtained for the M2 and M3 cycles. When $r_{m1}$, a, b, ϕ, B, L, ${\dot{m}}_{f}$ and ${\bar{S}}_{p}$ are known, $T_{3}$, $P_{3}$ and $T_{4M1}$ can be obtained by combining Equations (16), (33), (40) and (41) for the M1 cycle. The corresponding values for the Otto and Atkinson cycles can be obtained by making $r_{m1}=1$ and $r_{m1}=\frac{T_{4A}}{T_{1}}$, respectively. $T_{3M2}$, $T_{3M3}$, $P_{3M2}$, $P_{3M3}$, $T_{4M2}$ and $T_{4M3}$ for the M2 and M3 cycles can also be obtained by solving the corresponding equations described above. ${\dot{Q}}_{in}$ for the M1 cycle can be obtained by substituting $T_{2}$ and $T_{3}$ into Equation (16). ${\dot{Q}}_{out}$ can be obtained by substituting $T_{1}$, $T_{4M1}$ and $T_{5}$ into Equations (17)–(19). The lost power from friction can be obtained by substituting $P_{\max}$ and ${\bar{S}}_{p}$ into Equation (44). At last, the effective output power, the cycle efficiency and the power density can be computed by substituting ${\dot{Q}}_{in}$, ${\dot{Q}}_{out}$, $P_{f}$ and $V_{d\_M1}$ into Equations (46)–(48). The corresponding values can be obtained for the Otto, Atkinson and M2 and M3 cycles in a similar manner.

## 3. Assumptions and Simplifications

The objective of the paper is to qualitatively compare the energy losses and the performances of the cycles shown in Figure 1. The usability is to provide the knowledge about the influences of the cycle types on the ratios of various energy losses and the cycle performances; and the respective advantages and disadvantages of these cycles. These cycles are essentially irreversible considering the irreversibilities associated with the heat-transfer and friction; and the air and fuel rate, the heat-transfer rate and the lost power of friction are all related to the mean piston speed (temporal variable).

In [24], an irreversible parameter $I_{R}$ multiplied by the exhaust loss is introduced to quantify the internal irreversibility such as the fluid friction and combustion. It has been proven in the reference that an FTT model could qualitatively reproduce the behaviors and characterizations of real processes with some simplifications and assumptions such as a constant irreversibility parameter. In some other references such as [20], the internal irreversibility is described by a compression and an expansion efficiency. If the irreversible processes in the compression and expansion strokes are also marked in Figure 1, the cycle figures will appear too confused and difficult to identify. Therefore, in order to simplify the modeling process, we take the internal irreversible parameter or the compression and expansion efficiency as one considering that the objective of this paper is to perform a comparative study among the cycles, and the irreversibility in the compression and expansion processes has no obvious effect on the comparative analysis results.

At last, all compression and expansion processes are assumed adiabatic. All instantaneous values for cylinder pressure and charge temperature in the models are replaced by respective average values such as in Equation (22). The cylinder wall temperature is assumed constant in the heat-addition and heat-transfer processes. 

## 4. Results and Discussions

A cycle calculation has been performed in order to study and compare the performances of those cycles shown in Figure 1. Some model parameters are fixed in the calculation: $T_{1}=350\;K$, $T_{w}=400\;K$, (m˙am˙f)s=14.7, $Q_{LHV}=44,000\;(\mathrm{kJ}/\mathrm{kg})$, ${\dot{m}}_{a}=0.00082*{\bar{S}}_{p}\;(\mathrm{kg}/s)$, ϕ=0.9, ${\bar{S}}_{p}=8\;(m/s)$, B = L = 0.08 m; and all the other model parameters are calibrated to make the computed results approach a real engine. 

**Figure 2:**
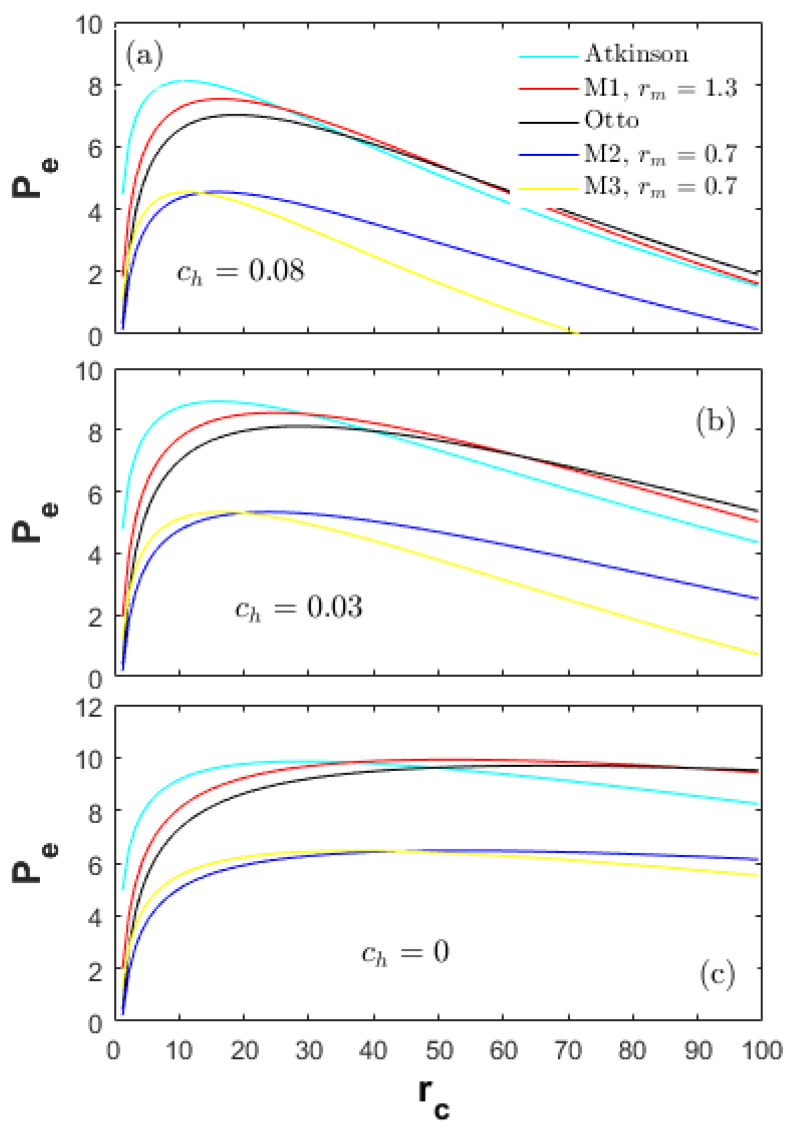
Effective power at different heat-transfer weights.

**Figure 3:**
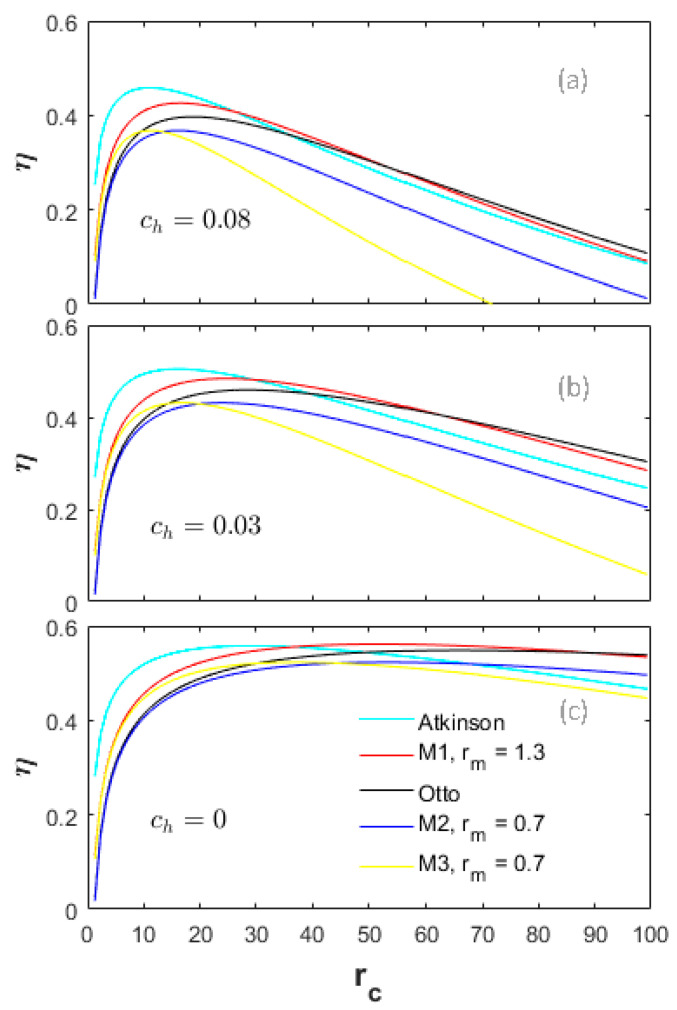
Energy efficiency at different heat-transfer weights.

**Figure 4:**
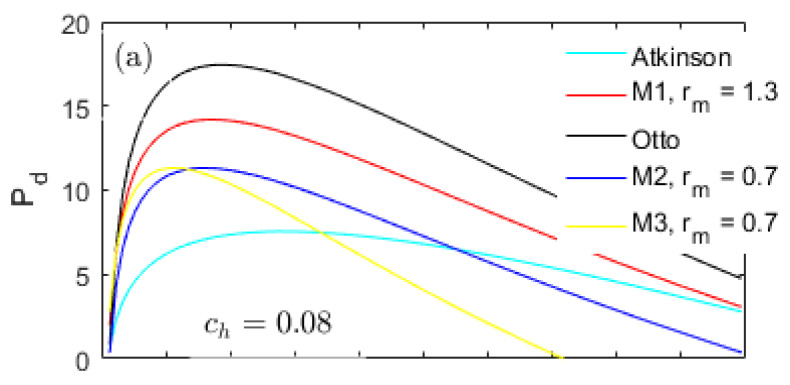

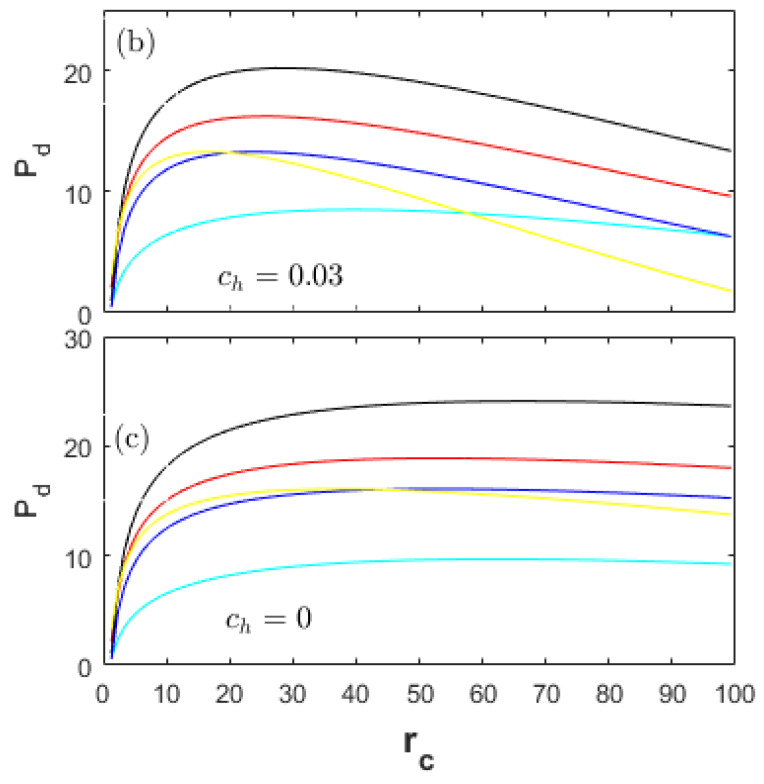
Power density at different heat-transfer weights.

A heat-transfer weight coefficient $c_{h}$ multiplied by the heat-transfer term in Equation (22), is defined to weigh the importance of the heat-transfer losses in the total input energy. Figure 2, Figure 3 and Figure 4 demonstrate the performances of the five thermodynamic cycles in Table 1 at different heat-transfer weights. The subfigures (a) in Figure 2, Figure 3 and Figure 4 show the performances of normal conditions with $c_{h}=0.08$; the subfigures (b) and (c) show the performances with lower heat-transfer weights in order to investigate the performance difference at different heat loss degrees.

Figure 2 demonstrates a comparison of the effective power ($P_{e}$) at different heat-transfer weights. The peak power of the Atkinson cycle is the maximum among the Otto, M1 and Atkinson cycles ($P_{e\_At}>P_{e\_M1}>P_{e\_Otto}$). In the low CR range, the $P_{e}$ of the Atkinson cycle is always higher than the other cycles. In the high CR range, the $P_{e}$ of the Otto cycle becomes the maximum. The $P_{e}$ of the M2 and M3 cycles are far less than the Otto, Atkinson and M1 cycles in the full CR range examined, which is because of the LIVC operation leading to the reduction of the net fuel amount in the cylinder. The peak $P_{e}$ of the M3 cycle is comparable to the M2 cycle, but the corresponding CR for the M3 cycle is lower, which may have some thermodynamic and dynamic advantages in a real engine such as NOx reduction and low vibration.

Figure 3 demonstrates a comparison of the energy efficiency (η) at different heat-transfer weights. The change tendency of the η for all cycles is basically the same as the effective power in Figure 2. In the low CR range, the η of the Atkinson cycle is always the maximum, and that of the M2 cycle is the lowest. In the high CR range, the η of the Otto cycle is the highest, while the one of the M3 cycle is the lowest. As the heat-transfer weights decrease, the η differences between the Otto, M1 and Atkinson cycles and the M2, M3 cycles decrease. As shown in Figure 3a, in the CR range less than 10, the η of the M3 cycle is higher than the Otto cycle. As the $c_{h}$ decreases from 0.08 to 0.03 to 0, the effective CR range that makes the η of the M3 cycle higher than the Otto cycle increases. Moreover, in the full CR range, the η of the M2 and M3 cycles are always lower than the M1 cycle; and the η of the M2 cycle are always lower than the Otto cycle.

Figure 4 shows a comparison of the power density ($P_{d}$) at different $c_{h}$ values. The peak power density for the Atkinson cycle is the lowest and even lower than the M2 and M3 cycles because of the largest displacement volume *V_d_*. In the full CR range examined, the power density for the Otto cycle is always the highest because of the modest effective power and *V_d_*. 

The power density for the M1 cycle falls between the Otto and Atkinson cycles; and always higher than the M2 and M3 cycles in the full CR range. Although the peak η of the Atkinson cycle is higher than the M1 cycle, its power density is too low. The Otto cycle has the highest power density, but a lower peak η than the M1 cycle. Moreover, as shown in Figure 2, the η of the M1 cycle are always higher than the M2 and M3 cycles in the full CR range. Therefore, the comprehensive performances of the M1 cycle are the optimum.

The performance results shown in Figure 2, Figure 3 and Figure 4 are together influenced by the cycle types and the energy losses. Investigation on the energy losses can provide guidance for improving the performances of a real engine. In this section, the parameters $R_{h}$, $R_{e}$ and $R_{f}$ are defined to denote the energy loss ratios of the heat-transfer, exhaust and friction in the total input energy, respectively.

**Figure 5:**
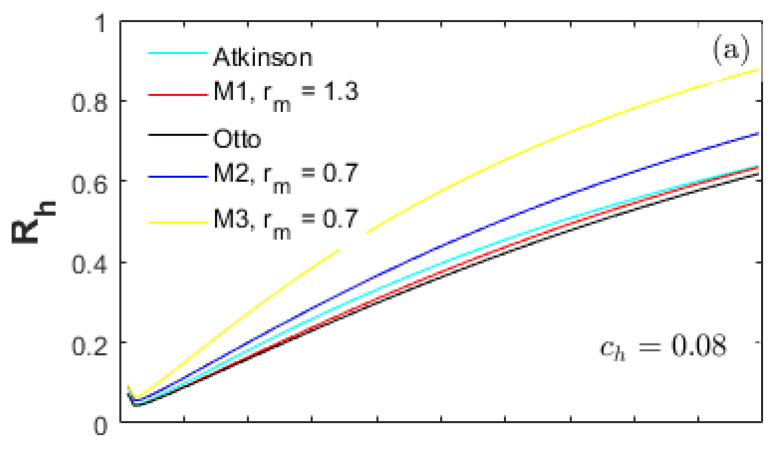

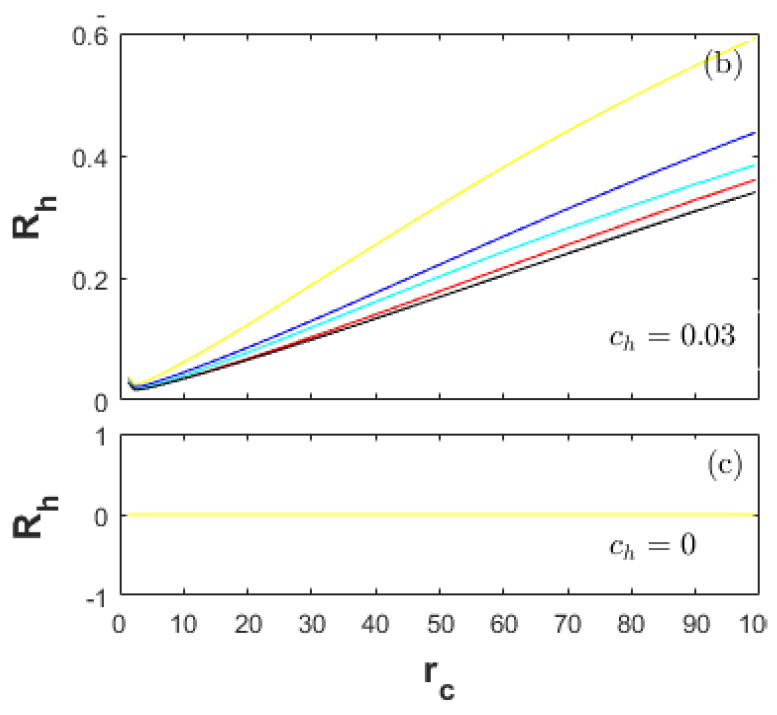
Ratios of heat-transfer loss for different heat-transfer weights.

Figure 5 is a comparison of the heat-transfer loss ratios ($R_{h}$) for all cycles examined. As shown in Figure 5a,b, the $R_{h}$ of the Otto cycle is the lowest; and the one for the M3 cycle is the highest in the full CR range examined ($R_{h\_Otto}<R_{h\_M1}<R_{h\_At}<R_{h\_M2}<R_{h\_M3}$).

**Figure 6:**
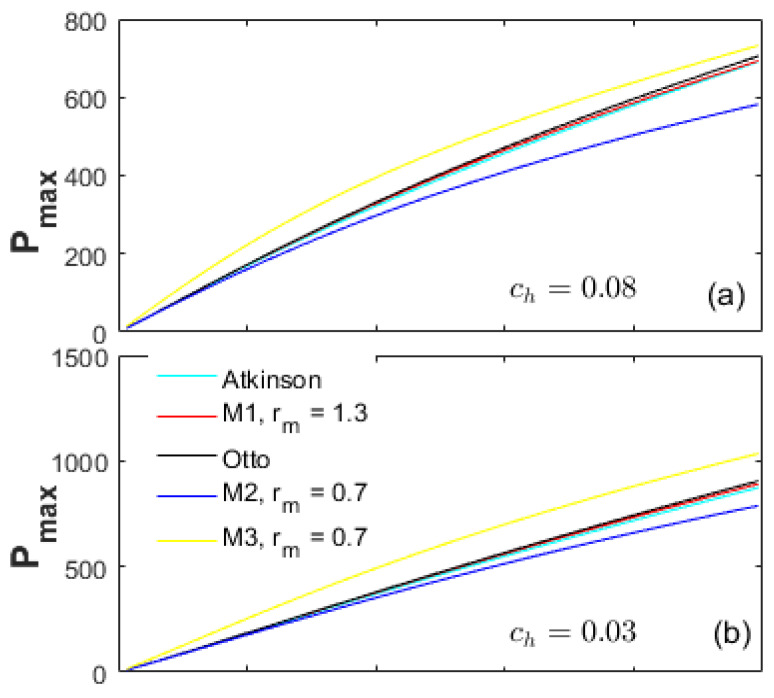

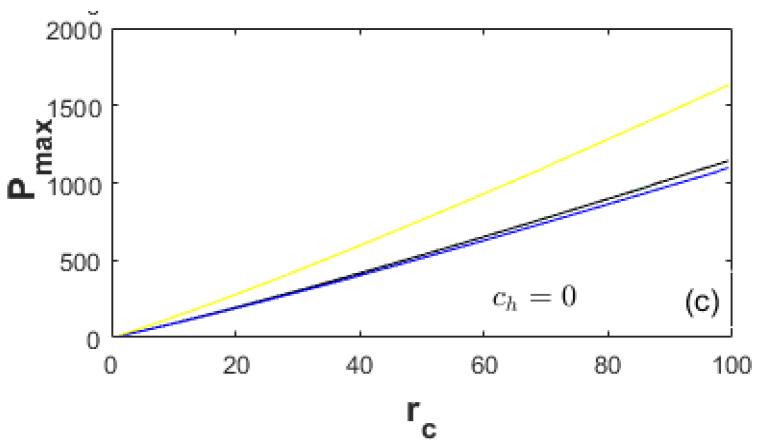
Peak cylinder pressure for different heat-transfer weights.

**Figure 7:**
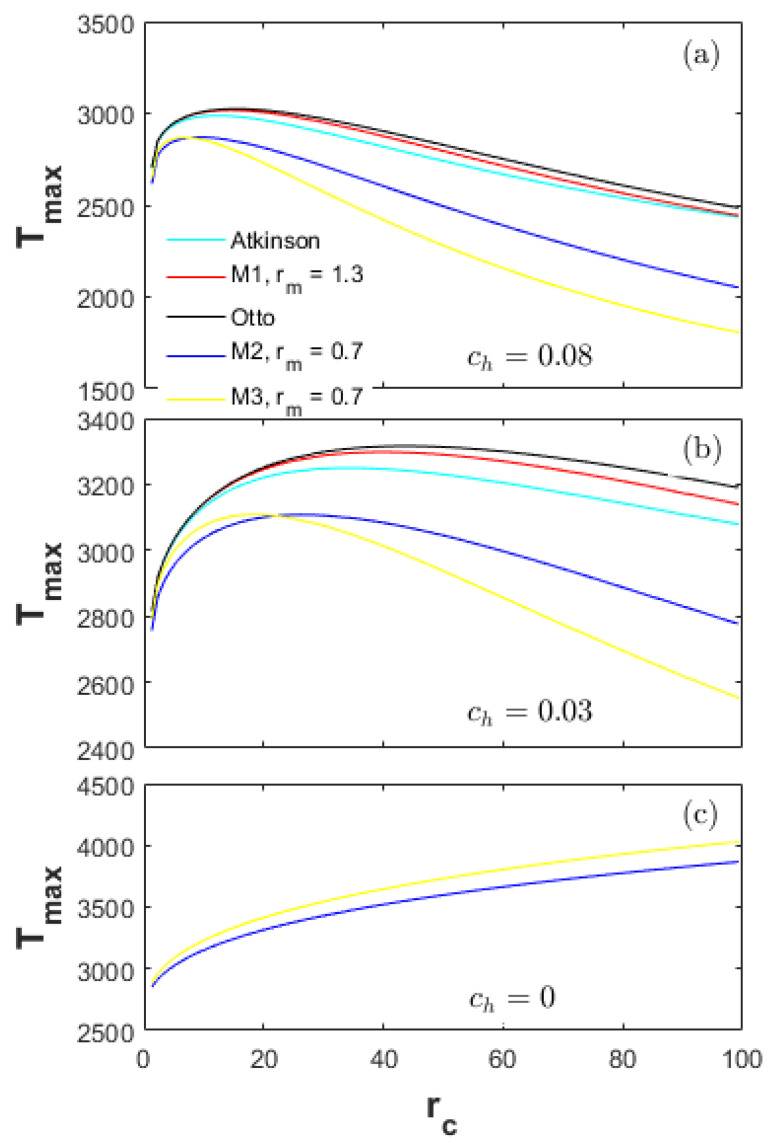
Peak in-cylinder gas temperature for different heat-transfer weights.

It is shown in Figure 6 that the peak cylinder pressure ($P_{\max}$) of the Otto cycle is slightly higher than the M1 and Atkinson cycles. A higher $P_{\max}$ increases the heat-transfer coefficient ($h_{c}$) and thus the heat-transfer loss. Moreover, the peak charge temperature ($T_{\max}$) of the Otto cycle is higher than the M1 and Atkinson cycles ($T_{\max\_Otto}>T_{\max\_M1}>T_{\max\_At}$), as shown in Figure 7. The high $T_{\max}$ can lead to more heat-transfer loss and a high $R_{h}$. As a result, the only reason leading to the high $R_{h}$ for M1 and Atkinson cycles is a bigger displacement volume $V_{d}$. A bigger $V_{d}$ can increase the $h_{c}$ (Equations (23) and (24)) and thus the heat-transfer loss. Therefore, the $R_{h}$ of the Atkinson cycle is higher than the M1 and Otto cycles ($R_{h\_Otto}<R_{h\_M1}<R_{h\_At}$).

As shown in Figure 5a,b, the $R_{h}$ for the M3 cycle is higher than the M2 cycle in the full CR range, which is because of the obviously higher in-cylinder mean pressure ($P_{2}$, $P_{\max}$) increasing the $h_{c}$ for the M3 cycle. Furthermore, in the full CR range, the $R_{h}$ of the M2 cycle is higher than the Otto, M1 and Atkinson cycles even though the ($P_{2}$, $P_{\max}$) and ($T_{2}$, $T_{\max}$) of the M2 cycle are lower. The major reason is the less net fuel amount in the cylinder for the M2 cycle because of the LIVC operation. As a result, the $R_{h}$ of the M2 cycle is higher even with the same amount of heat loss as the Otto, M1 and Atkinson cycles. 

**Figure 8:**
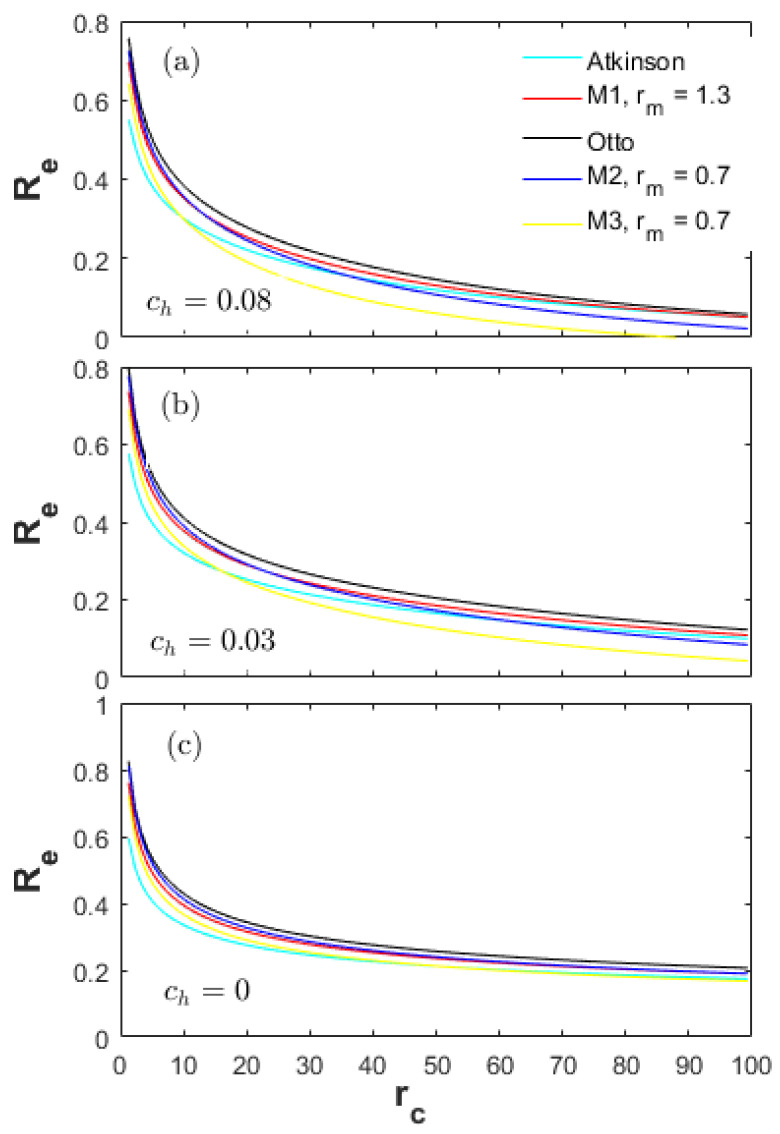
Ratios of exhaust loss for different heat-transfer weights.

Figure 8 is a comparison of the exhaust loss ratios ($R_{e}$) for all cycles examined. The $R_{e}$ is primarily influenced by the charge temperature at the end of the expansion stroke. Therefore, the expansion ratio ($r_{e}$) is the determining factor of the $R_{e}$. The bigger the $r_{e}$ is, the lower the charge temperature at the end of the expansion stroke is. The $R_{e}$ of the Atkinson cycle is the lowest ($R_{e\_At}<R_{e\_M1}<R_{e\_Otto}$) among the Otto, M1 and Atkinson cycles. The $R_{e}$ of the M3 cycle is obviously lower than the M2 cycle. Moreover, the $R_{e}$ of the M3 cycle is lower than the Atkinson cycle in the high CR range and lower than the M1 and Otto cycles in the full CR range even with a lower net fuel amount in the cylinder, which is because of the high $r_{e}$ and the low $T_{\max}$.

**Figure 9:**
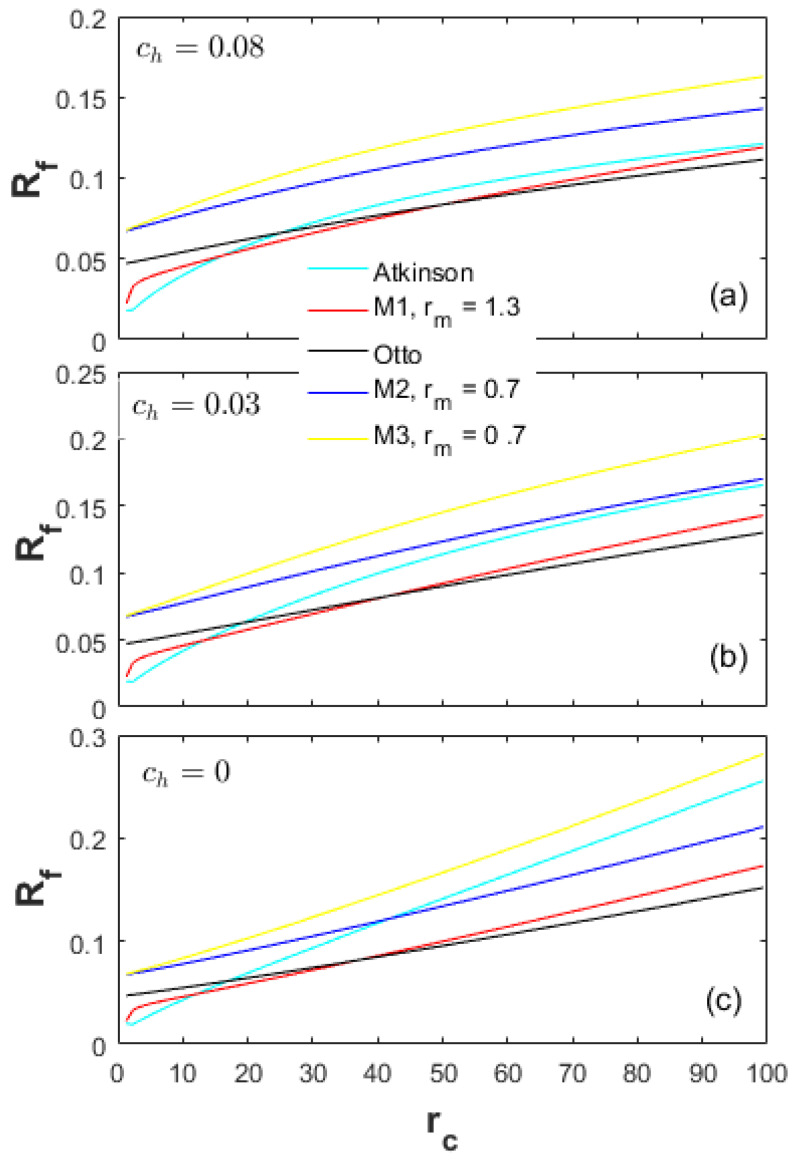
Ratios of friction loss for different heat-transfer weights.

Figure 9 shows a comparison of friction loss ratios ($R_{f}$) for all cycles examined. According to Equations (42)–(44), the following equations can be achieved:

(49) $$P_{f\_b}=\left(\frac{a\pi }{16\times 10^{3}}\right)P_{\max}L,$$

(50) $$P_{f\_V}=\left(\frac{bA_{eff}{\bar{S}}_{p}^{2}\pi }{16\times 10^{3}}\right)\frac{1}{L}.$$

In the low CR range, the $P_{f\_V}$ dominates. According to Equation (50), the $R_{f}$ of the Atkinson cycle is the lowest ($R_{f\_At}<R_{f\_M1}<R_{f\_Otto}$) because of having the longest expansion stroke. As the CR increases, the $L_{At}$ increases fast, and $P_{f\_b}$ is gradually dominating $R_{f}$. Thus, the $R_{f}$ of the Atkinson cycle is the highest in this range ($R_{f\_At}>R_{f\_M1}>R_{f\_Otto}$). As shown in Figure 6, the $P_{\max}$ of the Atkinson cycle is slightly lower than the M1 and Otto cycles in the full CR range. According to Equation (49), the reason for the highest $R_{f}$ of the Atkinson cycle can only be the biggest expansion stroke increasing the boundary friction losses. The change characteristics of the M1 cycle are similar to the Atkinson cycle. 

As shown in Figure 6, the $P_{\max}$ of the M3 cycle is greatly higher than the M2 cycle. According to Equation (49), the high $P_{\max}$ leads to a high $P_{f\_b}$. The $P_{f\_V}$ of the M2 and M3 cycles is equal. As a result, the $R_{f}$ of the M3 is obviously higher than the M2 cycle in the full CR range.

It is shown in Figure 9a,b that the $R_{f}$ of the M2 cycle is always higher than the Otto, M1 and Atkinson cycles in the full CR range. As shown in Figure 6, the $P_{\max}$ of the M2 cycle is lower than the Otto, M1 and Atkinson cycles. The expansion strokes (L) of the M2 and Otto cycles are also the same. Therefore, the $P_{f\_M2}$ is less than the Otto cycle, and the reason for the higher $R_{f}$ of the M2 cycle is the less net fuel amount in the cylinder. As a result, the load (more fuel amount injected) can also be raised in order to reduce the $R_{f}$ and improve the mechanical efficiency in a real engine. 

As shown in Figure 9, the difference between $R_{f\_At}$ and $R_{f\_M2}$ gradually decreases as $c_{h}$ decreases in the high CR range. $R_{f\_At}$ is even higher than $R_{f\_M2}$ as the $c_{h}=0$. Reducing the $c_{h}$ until zero, a part of the energy from the heat-transfer loss is converted to the effective power output; and another part is converted to the exhaust energy. In this situation, the Atkinson cycle must have a big expansion stroke in order to expand to the barometric pressure and temperature, leading to the fast increment of the $P_{f\_b}$ (Equation (49)). 

The performance results of the thermodynamic cycles are together determined by the ratios of the energy losses ($R_{h},R_{e},R_{f}$) and the cycle types. As an example of the Atkinson cycle, in the low CR range, the decrement of the $R_{e}$ + $R_{f}$ of the Atkinson cycle is larger than the increment of the $R_{h}$ compared to the M1 and Otto cycles. The efficiency of the Atkinson cycle is obviously higher and reaches the maximum in this range. The advantage of the $R_{e}$ of the Atkinson cycle gradually decreases as the CR further increases; and the $R_{f}$ of the Atkinson cycle begins higher than the M1 and Otto cycle. The increment of the $R_{f}$ + $R_{h}$ of the Atkinson cycle is higher than the decrement of the $R_{e}$; and the energy efficiency is lower than the M1 and Otto cycles. Therefore, the optimum CR ranges for the efficiency and effective power of the Atkinson, M1 and Otto cycles are different. 

The influence characteristics of the energy losses on the efficiency of the M2 and M3 cycles are similar to the Atkinson cycle. As shown in Figure 8, the $R_{e}$ of the M2 cycle is less than the Otto cycle in the full CR range; and even less than the M1 and Atkinson cycle in the high CR range. However, the $R_{f}$ and $R_{h}$ of the M2 cycle are obviously higher than the Otto, M1 and Atkinson cycle in the full CR range. The increment of the $R_{f}$ + $R_{h}$ of the M2 cycle is higher than the decrement of the $R_{e}$. Thus, the efficiency of the M2 is lower than the Otto, M1 and Atkinson cycles. The $R_{e}$ of the M3 cycle is greatly lower than the other cycles; and the obviously higher $R_{f}$ and $R_{h}$ of the M3 cycle discount the advantage of the $R_{e}$. Therefore, in a real engine, a high load (more fuel amount injected) under specific engine speed and equivalence ratio generally means a high energy efficiency.

**Figure 10:**
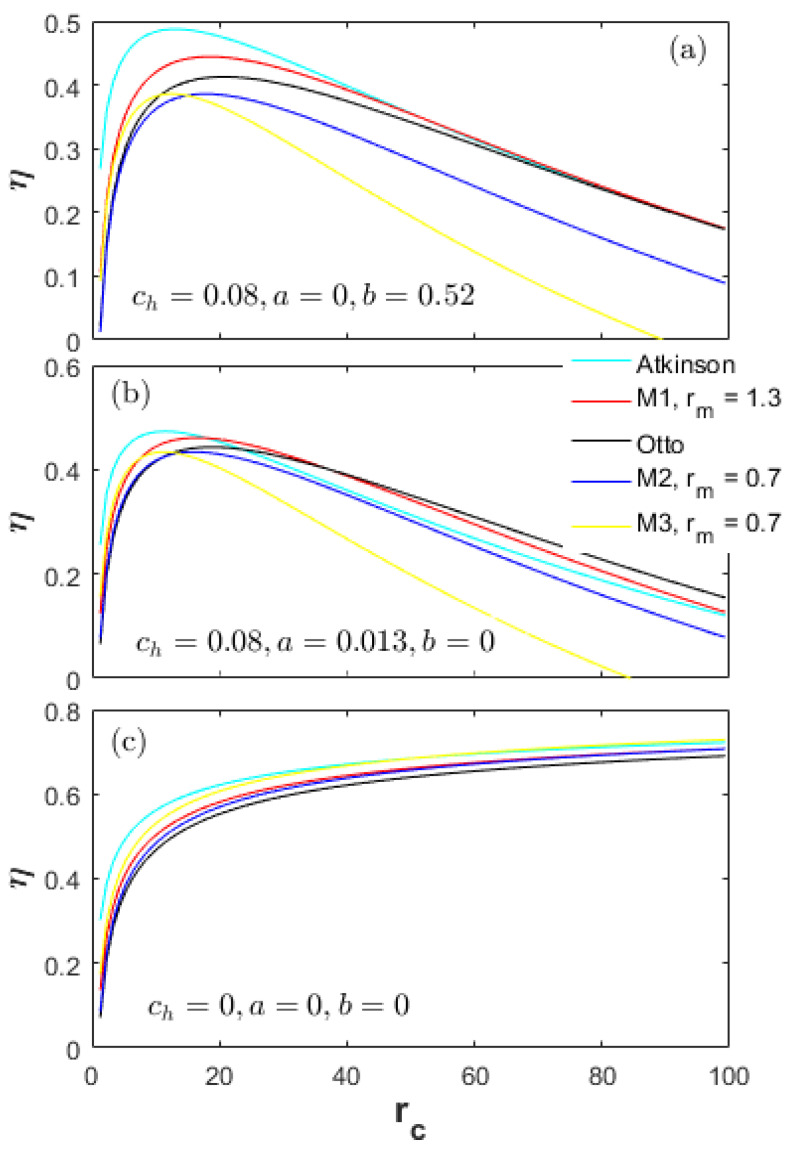
Energy efficiency for different friction factors.

**Figure 11:**
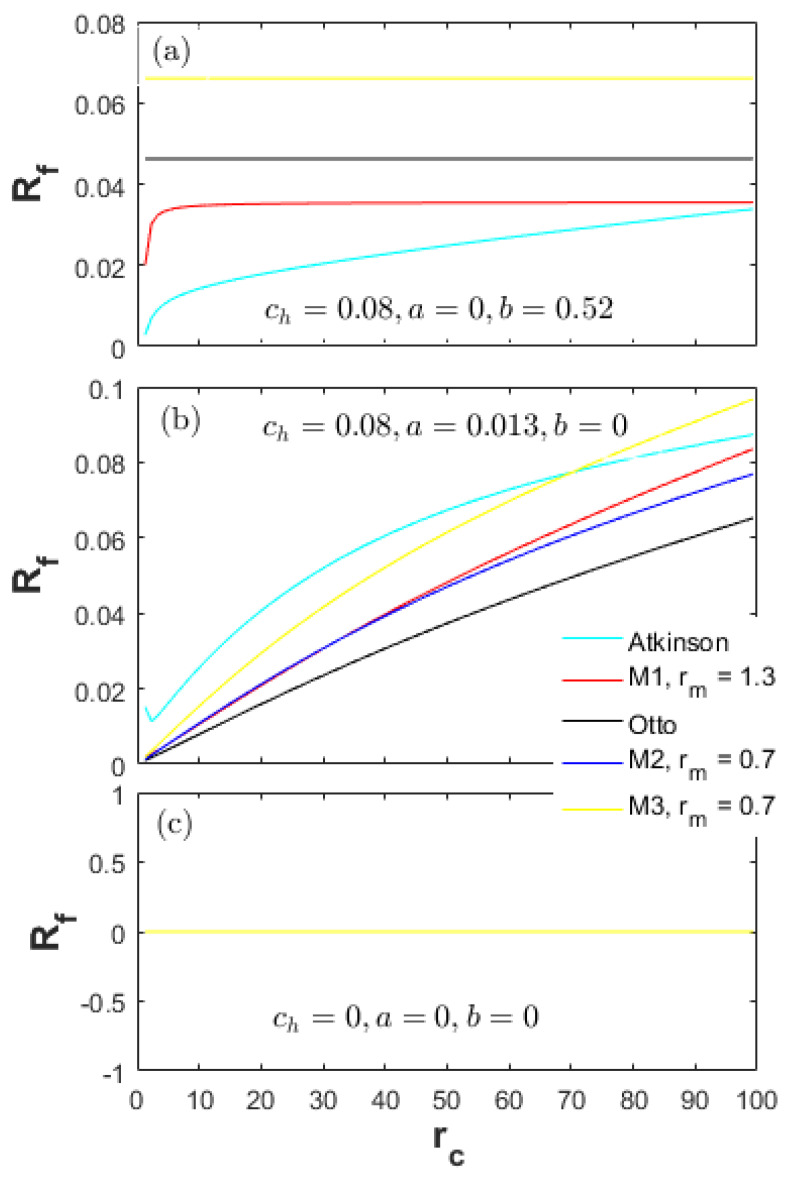
Ratios of friction loss for different friction factors.

Figure 10 and Figure 11 demonstrate the energy efficiency and friction loss of the thermodynamic cycles in Table 1 at different friction factors and without heat loss ($c_{h}=0$). The change characteristics of the effective power ($P_{e}$) and power density ($P_{d}$) are similar to the energy efficiency (η) and that in Figure 4, respectively. Additionally, the results of the $R_{e}$, $R_{h}$, $T_{\max}$ and $P_{\max}$ ($c_{h}=0.08$) with no relation to the friction factors have been presented in Figure 5, Figure 6, Figure 7 and Figure 8. These results are not presented here again.

As shown in Figure 10a, in the full CR range, the energy efficiency of the Atkinson cycle is always higher than the M1 and Otto cycles in the case of no boundary friction (*a* = 0) ($\eta_{\_At}>\eta_{\_M1}>\eta_{\_Otto}$). As shown in Figure 10b, in the high CR range, the η of the Atkinson cycle is less than the M1 and Otto cycles with only the boundary friction (*b* = 0) ($\eta_{\_At}<\eta_{\_M1}<\eta_{\_Otto}$). It is worth noting that when there is only the boundary friction (*b* = 0), the η of the M2 cycle has less difference with the Atkinson cycle especially in the middle CR range.

The performance characteristics described above are primarily influenced by $R_{f}$. For the Otto, M1 and Atkinson cycles in the full CR range as shown in Figure 11a, the $R_{f\_Otto}$ is the highest and constant, while the $R_{f\_At}$ increases as the CR increases and is always less than the M1 and Otto cycles. Therefore, in the high CR range, the η of the Atkinson cycle are not as in Figure 3 and always higher than the M1 and Otto cycles. As shown in Figure 11b, the $R_{f\_At}$ is obviously higher than the M1 and Otto cycles; and even higher than the M3 cycle in the middle to low CR range. Therefore, the η of the Atkinson cycle sharply drop down as the CR increases. 

As shown in Figure 10c, the η of all the cycles monotonously increase as the CR increases; and approach the ideal values. Furthermore, the η of the M3 cycle is higher than the M1, M2 and Otto cycles in the full CR range; and even slightly higher than the Atkinson cycle in the high CR range. This is primarily the high expansion ratio of the M3 cycle reducing the $R_{e\_M3}$. Therefore, in a real engine, some new techniques can be adopted to greatly reduce the heat-transfer and friction losses in order to improve the performances of the thermodynamic cycle, especially the M3 cycle. For example, a ceramic cylinder liner can be used to avoid the heat-transfer loss. Novel pistons and piston rings [35], symmetric crankshafts, low tension piston rings and low viscosity lubricating oil can be used to reduce the friction loss. 

Moreover, the effect of the in-cylinder charge temperature on the combustion efficiency is not considered in Equation (32). As a result, the maximum energy efficiency of the M2 cycle is almost the same as the M3 cycle even though the effective CR of the M3 cycle is higher. In a real engine, the in-cylinder charge temperature at the end of compression stroke is higher for the M3 cycle; and higher combustion efficiency can be achieved. Therefore, in a real engine, the energy efficiency of the M3 cycle is certainly higher than the M2 cycle; and the usage of the M3 cycle is better.

## 5. Conclusions

The FTT models for an Otto cycle, an over-expansion Miller cycle (M1), an Atkinson cycle, an LIVC Miller cycle (M2) and an LIVC Miller cycle with CCR (M3) are first established. The irreversible losses from heat-transfer and friction are considered in the models. The change characteristics and comparisons of the performances ($P_{e},\eta ,P_{d}$) and energy losses ($R_{f},R_{e},R_{h}$) with respect to the CR, the different heat-transfer weights and friction factors and the cycle types are deeply investigated. Some important conclusions have been obtained.

(1) The optimum CR ranges for the efficiency and effective power of the Atkinson, M1 and Otto cycles are different. The peak $P_{e}$ and η of the Atkinson cycle are the maximum, but the $P_{d}$ is particularly low. In the full CR range, the comprehensive performances of the M1 cycle are the optimum.
(2) In the full CR range, the $P_{e}$ of the M2 and M3 cycles is always lower than the Otto, M1 and Atkinson cycles because of the less net fuel amount in the cylinder. The $P_{d}$ is basically higher than the Atkinson cycle; and the η of the M3 cycle is higher than the Otto cycle in the low CR range. The maximum η of the M2 and M3 cycles are nearly the same because the effect of the charge temperature on the combustion efficiency is not considered in this study.
(3) The comparative results of cycle performances are influenced together by the ratios of the energy losses ($R_{h},R_{e},R_{f}$) and the cycle types. A long expansion stroke (Atkinson or M1 cycles) or a large expansion ratio (M3 cycle) can reduce the $R_{e}$, but has an adverse effect on the $R_{h}$ and $R_{f}$. In any CR range, the cycle performances improve if the advantages in the ratios of energy losses exceed the disadvantages. A large displacement volume is always adverse for the $P_{d}$ especially for the Atkinson cycle. The less net fuel amount and the high $P_{\max}$ (M3 cycle) have significantly adverse effect on the $R_{h}$ and $R_{f}$ of the M2 and M3 cycles; and the η is always lower than the M1 and Atkinson cycles in the full CR range.
(4) When there is only the boundary or viscous friction, the change characteristics of the performances for the Otto, M1 and Atkinson cycles are different. When reducing the heat-transfer and friction to zero, the M3 cycle has an obvious advantage in the η. In a real engine, some techniques such as a ceramic cylinder liner can be adopted to greatly reduce the heat-transfer and friction losses in order to improve the performances of thermodynamic cycles, especially the M3 cycle.

## Figures and Tables

**Table 1:** The cycles examined.

Cycle Names	Otto Cycle	Over-Expansion Miller Cycle	Atkinson Cycle	LIVC Miller Cycle	LIVC Miller Cycle with CCR
Abbreviations	Otto	M1	Atkinson	M2	M3
Process	1-2-3-4O-1	1-2-3-4M1-5-1	1-2-3-4A-1	1-1L-2M2-3M2-4M2-1	1-1L-2M3-3M3-4M3-1

## Nomenclature

NVH	Noise Vibration Harshness
ACE	Atkinson cycle engine
MP	maximum dimensionless power
MPD	maximum dimensionless power density
MEF	maximum thermal efficiency
TDC	top dead center
CR	compression ratio
$r_{m2}$	ratio of two compression ratios (-)
$m_{c}$	mass rate of intake charges (kg/s)
$r_{c}$	compression ratio (-)
$p_{e}$	effective power (kW)
*T*	temperature (K)
$p_{d}$	power density (kW/L)
$c_{v}$	constant volume specific heat (kJ/kg·K)
*N*	engine rotating speed (r/s)
$Q_{LHV}$	low heat value of fuel (kJ/kg)
$\dot{Q}$	rate of heat added (kJ/s)
$A_{cc}$	surface area of combustion chamber (m^2^)
*p*	instantaneous cylinder pressure (bar)
*a*, *b*, *C*	constants (-)
MCE	Miller cycle engine
FTT	finite-time thermodynamics
OMC	Otto–Miller Cycle
LIVC	late intake valve closure
CCR	constant compression ratio
FMEP	friction mean effective pressure
*R*	ratio of energy loss (-)
$r_{e}$	expansion ratio (-)
*B*	cylinder bore (m)
*L*	stroke length (m)
$r_{m1}$	ratio of two expansion ratios (-)
*V*	volume (m^3^)
$C_{p}$	constant pressure specific heat (kJ/kg·K)
${\bar{S}}_{p}$	mean piston speed (m/s)
$R_{g}$	gas constant (kJ/kg·K)
$h_{c}$	convective heat-transfer coefficient (Kj/m^2^·K·s)
*w*	average in-cylinder charge velocity (m/s)
$A_{p\_eff}$	effective area of piston skirt (m^2^)
$P_{f}$	friction lost power (kW)
*P*	cylinder pressure (bar), power (kW)
**Greek Letters**	
*η*	effective efficiency (-)
ϕ	equivalence ratio (-)
**Subscripts**	
M2	M2 cycle
M3	M3 cycle
T	Total
c	charges
in	input
tr	transfer
r	reference
m	mean
d	displacement, density
h	heat-transfer
max	maximum
M1	M1 cycle
f	fuel, friction
a	air
out	output
m	motored
com	combustion
w	wall
at	Atkinson
e	exhaust, effective
